# Advances in the detection of biomarkers for ischemic stroke

**DOI:** 10.3389/fneur.2025.1488726

**Published:** 2025-02-24

**Authors:** Ying Liang, Juan Chen, Yue Chen, Yaoyao Tong, Linhao Li, Yuan Xu, Shimin Wu

**Affiliations:** ^1^Center for Clinical Laboratory, General Hospital of the Yangtze River Shipping, Wuhan Brain Hospital, Wuhan, Hubei, China; ^2^Center for Clinical Laboratory, Wuhan Hospital of Traditional Chinese Medicine, Wuhan, Hubei, China

**Keywords:** stroke, biomarkers, inflammatory factors, axonal injury markers, clinical testing

## Abstract

Ischemic stroke is a leading cause of mortality and morbidity globally. Prompt intervention is essential for arresting disease progression and minimizing central nervous system damage. Although imaging studies play a significant role in diagnosing ischemic stroke, their high costs and limited sensitivity often result in diagnostic and treatment delays. Blood biomarkers have shown considerable promise in the diagnosis and prognosis of ischemic stroke. Serum markers, closely associated with stroke pathophysiology, aid in diagnosis, subtype identification, prediction of disease progression, early neurological deterioration, and recurrence. Their advantages are particularly pronounced due to their low cost and rapid results. Despite the identification of numerous candidate blood biomarkers, their clinical application requires rigorous research and thorough validation. This review focuses on various blood biomarkers related to ischemic stroke, including coagulation and fibrinolysis-related factors, endothelial dysfunction markers, inflammatory biomarkers, neuronal and axonal injury markers, exosomes with their circular RNAs and other relevant molecules. It also summarizes the latest methods and techniques for stroke biomarker detection, aiming to provide critical references for the clinical application of key stroke biomarkers.

## 1 Introduction

Stroke remains one of the leading causes of disability and mortality worldwide. According to the World Health Organization (WHO), stroke is the second leading cause of death globally, accounting for approximately 5.5 million deaths annually, with about half of the survivors experiencing long-term disability (1, 2). Among these, ischemic stroke constitutes approximately 80% of all stroke cases, primarily caused by the obstruction of cerebral blood vessels, leading to ischemia and hypoxia of brain tissue, and resulting in irreversible neuronal damage (3). Early diagnosis and timely treatment are crucial for reducing the disability and mortality rates among ischemic stroke patients (4, 5). Currently, the primary clinical treatment strategy for ischemic stroke is intravenous thrombolysis within the “golden window” to restore blood perfusion. However, some patients present with subtle or atypical symptoms, and the specificity of imaging-based assessments remains limited, leading to misdiagnosis, missed diagnoses, and delays in clinical decision-making. Therefore, there is an urgent need to establish novel laboratory-based rapid auxiliary diagnostic strategies (6).

Biomarkers are defined as measurable indicators that objectively reflect normal or pathological physiological processes or predict and assess responses to therapeutic interventions. In the context of ischemic stroke, biomarkers can provide insights into the pathophysiological mechanisms triggered by cerebrovascular occlusion, including inflammation, oxidative stress, and neuronal injury. They not only facilitate early diagnosis and subtype differentiation but also serve as crucial tools for disease assessment, prognosis prediction, and individualized therapeutic decision-making (7). Compared to traditional imaging-based diagnostics, biomarker detection in blood or other bodily fluids is generally more cost-effective, technically less complex, and offers the potential for dynamic monitoring, making it an indispensable tool in clinical practice (8).

The study of stroke biomarkers has been extensively discussed in multiple reviews, yet existing literature often lacks a comprehensive evaluation of their clinical applications and associated challenges. This review aims to systematically summarize the currently identified biomarkers for ischemic stroke, evaluate their research status and detection methodologies, and explore unresolved critical issues and future research directions.

## 2 Pathogenesis of ischemic stroke

Acute ischemic stroke (AIS) primarily results from atherosclerosis, cardioembolism, small vessel disease, and other rare causes, such as hypercoagulable states, arterial dissection, and genetic disorders. Among these, atherosclerosis is the most common mechanism, characterized by lipid deposition, chronic inflammation, and endothelial dysfunction, leading to the formation of atherosclerotic plaques. Plaque rupture can trigger platelet aggregation and coagulation cascade reactions, ultimately leading to thrombosis and acute occlusion of cerebral arteries, resulting in symptoms such as cerebral ischemia, neuronal necrosis, and functional impairment (9).

The pathogenesis of ischemic stroke involves multiple pathological processes, among which thrombosis is a key mechanism (10). The rupture of atherosclerotic plaques exposes the subendothelial matrix, leading to platelet activation and coagulation factor recruitment, thereby triggering the coagulation cascade. This process results in thrombus formation and vascular occlusion. The coordinated interaction between platelet activation and fibrin formation is a critical pathological event, along with abnormal activation of the coagulation system. Glial cells also play a crucial role in cerebral ischemia (11). The activation of microglia and astrocytes exhibits spatiotemporal specificity. In the early phase, microglia predominantly exhibit a pro-inflammatory phenotype (M1), concurrently inducing astrocytes to transition into a pro-inflammatory subtype (A1). As the disease progresses, microenvironmental signals drive these cells toward an anti-inflammatory phenotype (M2 and A2), thereby facilitating tissue repair and neuronal functional recovery. During neuronal injury, astrocytes release glial fibrillary acidic protein (GFAP), which is closely associated with the severity of neuronal damage.

**Figure 1:**
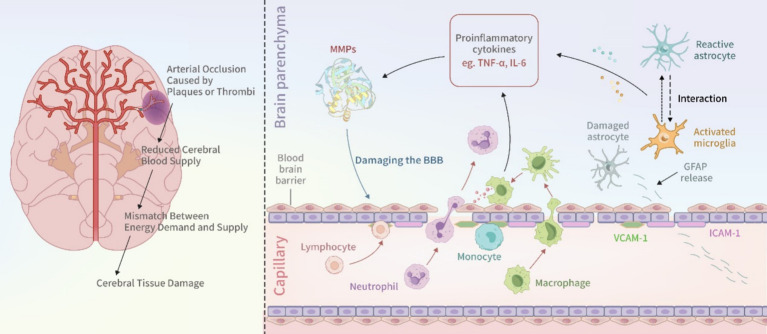
Pathogenesis of ischemic stroke.

Within hours following a stroke, the ischemic brain tissue rapidly initiates an inflammatory response. Perivascular microglia and macrophages release various cytokines, including tumor necrosis factor-alpha (TNF-*α*) and interleukin-6. Neutrophil infiltration occurs within minutes of ischemic stroke onset and peaks between 24 and 72 h. Within 48 h, monocytes and lymphocytes are also recruited to the brain (12). Subsequently, activated microglia, macrophages, and infiltrating leukocytes release additional inflammatory mediators, including TNF-*α* and interleukins (IL-1β, IL-6), thereby initiating a sustained inflammatory response through the secretion of IL-8 and IL-6. This inflammatory cascade leads to increased levels of fibrinogen and C-reactive protein (CRP), as well as the upregulation of adhesion molecules, including members of the immunoglobulin superfamily such as intercellular adhesion molecule-1 (ICAM-1) and vascular cell adhesion molecule-1 (VCAM-1) (13). Additionally, pro-inflammatory factors enhance matrix metalloproteinase (MMP) activity, further disrupting the blood–brain barrier (BBB), exacerbating cerebral edema, and promoting neuronal damage. Notably, MMP-9 levels significantly increase following stroke, contributing to extracellular matrix degradation and leukocyte infiltration, thereby amplifying the inflammatory response (Figure 1).

Thus, the pathogenesis of ischemic stroke involves multiple pathological pathways and bioactive molecules, many of which can serve as diagnostic and prognostic biomarkers. Based on their respective pathophysiological roles, these biomarkers can be broadly categorized into coagulation and fibrinolysis-related factors, endothelial dysfunction markers, inflammatory biomarkers, neuronal and axonal injury markers, and other relevant molecules (Table 1).

**Table 1:** Biomarkers for stroke detection.

Mechanism	Biomarker	Sample size	Clinical significance	Current clinical validation stage	References
Coagulation and fibrinolysis-related factors	Prothrombin, plasminogen, fibrinogen alpha-chain, and histidine-rich glycoprotein	95	Diagnostic	Prothrombin and plasminogen have been widely used for routine testing, while others are still under exploratory research	(19)
	Ceruloplasmin, *α*-1-antitrypsin (SERPINA1), vWF, and coagulation factor XIII B chain (F13B)	60	Diagnostic		(20)
Endothelial dysfunction-related biomarkers	ICAM-1, VCAM-1	131	Diagnostic	Exploratory research stage	(23)
	ICAM-1	69	Diagnostic		(24)
		286	Prognosis		(25)
		118	Prognosis		(27)
		113	Prognosis		(28)
	VCAM-1	38	Prognosis		(29)
	VWF	90	Diagnostic		(30)
		40	Diagnostic		(31)
Inflammatory markers	hsCRP	9,438	Prognosis	MMP-9 and HGMB-1are still under exploratory research, while others have been widely used for routine testing.	(35)
	IL-6, IL-1 *β*, IL-8, TNF-*α*, and hsCRP	680	Diagnosis and prognosis		(36)
	IL-6, CRP, WBC	138	Prognosis		(37)
	hsCRP, IL-6, Ferritin, ESR, and WBC	321	Prognosis		(38)
	hs-CRP,IL-6,TNF-α	588	Diagnostic and Prognostic		(39)
	MMP-9	60	Diagnostic		(41)
		62	Diagnostic		(42)
		3,186	Prognosis		(43)
	HGMB-1	183	Prognosis		(46)
		42	Prognosis		(47)
		544	Prognosis		(48)
Neuronal and axonal injury markers	GFAP, UCH-L1	251	Diagnostic	Exploratory research stage	(21)
	RBP-4, NT-proBNP, and GFAP	189	Diagnostic		(52)
	GFAP	155	Diagnostic		(53)
	GFAP and NT-proBNP	200	Diagnostic		(55)
	NfL	595	Prognosis		(55)
		211	Prognosis		(56)
		30	Prognosis		(57)
	MBP	83	Diagnostic		(60)
Exosomes and their circular RNA	lnc-CRKL-2, lnc-NTRK3-4, RPS6KA2-AS1and lnc-CALM1-7	200	Diagnostic	Exploratory research stage	(59)
	circFUNDC1	30	Diagnostic		(60)
	Exosomal circ_0043837 and circ_0001801	621	Diagnostic		(61)
	Exosomal Mir-134	10,172	Diagnostic		(62)
	Exosomal microRNA-21-5p and microRNA-30a-5p	167	Diagnostic		(63)
	exo-lnc_000048, exo-lnc_001350 and exo-lnc_016442	602	Diagnostic		(64)
Others	NETs	243	Prognosis	Exploratory research stage	(70)
		95	Diagnostic		(17)
		54	Prognosis		(71)
		101	Prognosis		(72)
		235	Prognosis		(73)

It is important to note that the pathophysiological processes of ischemic stroke exhibit distinct temporal dynamics. Specifically, in the hyperacute phase (<6 h), acute phase (6–72 h), and subacute phase (>72 h), different blood biomarkers display varying sensitivities and specificities (14). Therefore, the temporal dependency of biomarker expression must be carefully considered in both research and clinical applications to achieve more precise diagnostic and prognostic evaluations. For example, IL-6 levels begin to rise within hours after stroke onset, and persistently elevated IL-6 levels in the subacute phase may indicate ongoing inflammatory injury (15, 16). Similarly, GFAP is released progressively within 12 h post-stroke, making it a valuable biomarker for predicting intracranial pathology in both the hyperacute and acute phases. Furthermore, neutrophil extracellular traps (NETs) exhibit rapid fluctuations following thrombolysis or mechanical thrombectomy, providing insights into reperfusion status and aiding in the identification of futile recanalization (17).

## 3 Biomarkers associated with ischemic stroke

### 3.1 Coagulation and fibrinolysis-related factors

The coagulation and fibrinolysis systems are pivotal in the pathophysiology of stroke. Detecting related factors is vital for the early diagnosis and treatment of stroke. Biomarkers such as thrombin-antithrombin complex (TAT), tissue plasminogen activator inhibitor complex (t-PAIC), activated partial thromboplastin time (APTT), prothrombin time (PT), fibrinogen (FIB), D-dimer, and fibrin degradation products (FDP) reflect dynamic changes in thrombosis and fibrinolytic activity, aiding clinical decision-making. Elevated D-dimer levels indicate active fibrinolysis, commonly used to assess thrombus burden and prognosis in stroke patients. Ohara et al. highlighted that serum D-dimer assists in diagnosing cryptogenic stroke and secondary prevention, with continuous monitoring enhancing the efficacy of antithrombotic treatment in cryptogenic stroke (18). Lee et al., through proteomics, identified four candidate biomarkers—prothrombin, plasminogen, fibrinogen alpha chain, and histidine-rich glycoprotein—with AUC values over 0.9, confirming their diagnostic value related to coagulation mechanisms (19). Misra et al. used SWATH-MS-based proteomics to identify ceruloplasmin, *α*-1-antitrypsin (SERPINA1), von Willebrand factor (vWF), and coagulation factor XIII B chain (F13B) as effective biomarkers for distinguishing total stroke, ischemic stroke, and intracerebral hemorrhage (ICH) from healthy controls (20). Bioinformatics suggested common pathways in stroke cases, including complement and coagulation cascades, platelet degranulation, immune processes, and acute phase reactions (21).

### 3.2 Endothelial dysfunction-related biomarkers

Endothelial dysfunction can lead to dysregulation of vascular endothelial cell function, further exacerbating cerebral blood flow reduction and tissue damage.

#### 3.2.1 ICAM-1 and VCAM-1

ICAM-1 and VCAM-1 belong to the immunoglobulin superfamily and are primarily expressed on the surface of endothelial cells. Following ischemic injury, elevated levels of pro-inflammatory cytokines induce the expression of ICAM-1 and VCAM-1 in the endothelial cells of the blood–brain barrier (BBB), thereby mediating neuroinflammation (22). Elevated levels of ICAM-1 and VCAM-1 have been detected in the blood and infarct regions of stroke patients (23). Studies suggest that ICAM-1 may serve as a potential prognostic biomarker for AIS. Nielsen et al. evaluated ICAM-1 levels in AIS patients and found that they were significantly elevated within <8 h of stroke onset, whereas S100B and E-selectin levels showed no significant changes (24). Additionally, Wang et al. reported that the sensitivity and specificity of serum ICAM-1 in predicting AIS were 74 and 76%, respectively (25). Moreover, the rs5498 polymorphism of ICAM-1 has been associated with an increased risk of ischemic stroke in Caucasian populations (26). Furthermore, the combined detection of ICAM-1 and CRP has been shown to predict the 3-month prognosis of AIS patients (27). However, some studies have failed to establish a significant correlation between soluble ICAM-1 and stroke prognosis (28). In contrast, VCAM-1 levels have been proposed as a predictor of stroke prognosis, although they do not correlate with infarct volume or disability severity (29). Overall, the diagnostic and prognostic value of ICAM-1 and VCAM-1 in ischemic stroke remains incompletely understood, necessitating further research and clinical validation.

#### 3.2.2 von Willebrand factor

VWF is a multimeric glycoprotein secreted by endothelial cells and megakaryocytes, primarily involved in platelet adhesion and blood coagulation. Sabbah et al. found that serum VWF levels were significantly elevated in patients with AIS compared to control groups. Elevated plasma VWF levels were observed in patients with acute ischemic atherosclerotic stroke, suggesting that serum VWF levels could serve as a biomarker for AIS, particularly for the atherosclerotic subtype (30).

Sharma et al., through proteomic studies, also identified VWF as useful in distinguishing total stroke, ischemic stroke, and intracerebral hemorrhage (ICH) from healthy controls. Changes in its concentration may lead to endothelial dysfunction and are associated with inflammation and endothelial dysfunction in AIS patients, making it a novel candidate protein (31). Steliga et al. also summarized that VWF could serve as a diagnostic biomarker for AIS (32). Baez et al. included 12 articles involving blood combinations of stroke protein biomarkers and proposed a new biomarker combination model (NR2 + GFAP + MMP-9 + VWF + S100β) involving VWF for the early diagnosis of ischemic stroke subtypes (33). Moreover, VWF is considered an effective biomarker for predicting the risk of death in ischemic stroke patients. Kawano et al. found that elevated VWF levels are an independent predictor of mortality within 1 year after stroke onset (34). Further research on these biomarkers and their roles in the pathophysiology of ischemic stroke is crucial for improving patient prognosis and developing targeted therapeutic strategies.

### 3.3 Inflammatory markers

C-reactive protein (CRP), as an acute-phase protein, serves not only as an indicator of systemic inflammation but also as a crucial biomarker for evaluating prognosis post-stroke. Elevated CRP levels are associated with poor outcomes in stroke patients and reflect the systemic inflammatory state (35). IL-6 plays a key role in immune regulation within the central nervous system, while TNF-α exacerbates brain tissue damage by inducing apoptosis and promoting inflammatory responses (36).

Lasek-Bal et al. reported that IL-6 levels on the first day after stroke could predict acute neurological and functional status, while increased CRP and leukocyte counts were associated with worse acute stroke prognosis (37). Reiche et al. studied two composite indices reflecting inflammation levels—INFLAM Index 1 (comprising the z-scores of hsCRP, IL-6, ferritin, ESR, and WBC) and INFLAM Index 2 (derived by subtracting the z-score of 25(OH)D from INFLAM Index 1 and adding the z-scores of iron and TSP). These indices demonstrated significant predictive value for AIS in both healthy volunteers and AIS patients, with AUC values of 0.851 and 0.870, respectively. They also identified redox imbalance related to IL-6 signaling as a potential target for preventing short-term mortality in AIS (38). Ma et al. developed diagnostic and prognostic models for ischemic stroke using inflammatory markers such as hs-CRP, IL-6, and TNF-*α*, which were validated in additional cohorts (39).

Matrix metalloproteinase-9 (MMP-9) plays a crucial role in degrading components of the extracellular matrix, activating pro-inflammatory cytokines, and compromising the integrity of the blood–brain barrier (BBB). The activation of M1-polarized microglia has been shown to upregulate MMP-9 expression, leading to BBB disruption and ischemic brain injury (40). Abdelnaseer et al. reported that serum MMP-9 levels within 24 h of stroke onset were significantly correlated with clinical stroke severity (41). Similarly, Weekman et al. demonstrated a positive association between MMP-9 levels and infarct volume, with the strongest correlation observed within the first 6 hours post-stroke. Notably, MMP-9 is considered the only biomarker capable of precisely predicting the final infarct volume, where higher MMP-9 expression is linked to larger infarct areas (42). Further research by Zhong et al. revealed that elevated serum MMP-9 levels in the acute phase of ischemic stroke were positively correlated with mortality and severe disability within 3 months post-stroke (43). These findings suggest that targeted inhibition of MMP-9 activity may serve as a promising therapeutic strategy to mitigate brain injury and improve stroke prognosis (44).

High mobility group box 1 (HMGB1) has been identified as a potential diagnostic and prognostic biomarker for ischemic stroke (45). A study by Tsukagawa demonstrated that serum and plasma HMGB1 levels were significantly elevated in patients with ischemic stroke (46). Moreover, Sapojnikova et al. reported a strong correlation between MMP-9 and HMGB1 levels in stroke patients, with both biomarkers closely associated with poor prognosis (47). Similarly, Shen et al. found that elevated serum HMGB1 levels served as a reliable predictor of AIS recurrence (48). However, it is important to note that HMGB1 exhibits a complex biphasic role in the pathogenesis and progression of ischemic stroke. In the hyperacute and acute phases (within 4–5 days post-stroke), HMGB1 functions as a pro-inflammatory mediator, exacerbating neuronal death and blood–brain barrier disruption. Conversely, in the late acute, subacute, and chronic phases (>3 weeks post-stroke), HMGB1 contributes to vascular remodeling and neurofunctional recovery (49).

### 3.4 Neuronal and axonal injury markers

Ischemic stroke leads to increased blood–brain barrier permeability and the release of neuronal and axonal injury biomarkers, such as GFAP, neurofilament light chain protein (NFL), and S100 proteins. These markers are rapidly released into the blood following brain tissue injury, with their levels accurately reflecting the extent of brain damage and providing crucial prognostic information. Luger et al. conducted a study to assess the diagnostic accuracy of serum GFAP and ubiquitin carboxy-terminal hydrolase L1 (UCH-L1) concentrations, measured using ELISA, in differentiating between acute cerebral hemorrhage and ischemic stroke. The results indicated that the area under the curve (AUC) for GFAP was 0.866, surpassing the 0.590 AUC for UCH-L1 (21). Bustamante et al. validated a panel of blood biomarkers, including RBP-4, NT-proBNP, and GFAP, which distinguished IS from ICH with moderate accuracy at 100% specificity (50). Kalra et al. tested the diagnostic accuracy of GFAP in a prospective cohort of stroke patients in India. Using the highly sensitive SIMOA technology, GFAP concentrations were measured within 12 h of admission in acute stroke patients. ROC analysis identified an optimal GFAP threshold of 0.57 μg/L for distinguishing intracerebral hemorrhage from ischemic stroke and stroke mimic conditions (AUC 0.871 [95% CI 0.810–0.933], *p* < 0.001) (51). Recently, a systematic review confirmed the high diagnostic accuracy of blood GFAP levels as a discriminative test for cerebral hemorrhage and ischemic stroke (52). Additionally, Lee et al. developed a time-resolved fluorescence lateral flow immunoassay (TRF-LFIA) utilizing europium nanoparticle (EuNP)-conjugated specific monoclonal antibodies targeting NT-proBNP and GFAP for simultaneous quantification. The combination of GFAP and NT-proBNP was determined to be the most effective biomarker pair for differentiating IS from HS based on an algorithm (53). In summary, the use of blood biomarkers, particularly GFAP, holds promise for the diagnosis and differentiation of ischemic stroke and cerebral hemorrhage. Further research is necessary to validate these findings and establish standardized protocols for measuring acute stroke biomarkers.

Neurofilament light chain (NfL) levels have been shown to be a robust biomarker in cerebrospinal fluid (CSF) for neuronal damage and neurodegeneration. Several studies have demonstrated the potential of NfL as a blood biomarker for ischemic stroke. Sanchez et al. conducted a systematic review and meta-analysis, including 19 studies that reported serum/plasma NfL values from a total of 4,237 different stroke patients, to evaluate the utility of blood NfL as a diagnostic, prognostic, and monitoring biomarker for stroke. They found that blood NfL levels varied significantly across three different time periods: acute (0–7 days), subacute (9–90 days), and chronic (>90 days) phases of stroke, with a sharp peak observed in the early subacute phase, 14 to 21 days post-stroke. Additionally, blood NfL can serve as a diagnostic biomarker for differentiating AIS from transient ischemic attacks and other cerebrovascular subtypes (54). Pedersen et al. included 595 ischemic stroke cases in their study to investigate the correlation between serum NfL concentrations at different time points post-stroke. They found that NfL could predict neurological and functional outcomes in both the acute phase (range 1–14 days, median 4 days) and the long-term (cases followed up after 3 months) (55). Uphaus et al. also highlighted NfL as a biomarker for predicting cerebrovascular function 90 days post-ischemic stroke (56). Barba et al. explored the relationship between serum NfL concentrations and clinical outcomes in patients with AIS, finding that patients with higher NfL levels showed less clinical improvement post-treatment. In patients with moderate to severe AIS, serum NfL levels were correlated with clinical and radiological scores at different time points and were predictive of short-term and intermediate-term clinical outcomes (57).

Myelin basic protein (MBP) is a membrane protein synthesized by oligodendrocytes that plays a crucial role in stabilizing myelin structure and is highly specific to neural tissue. In cases of brain injury, myelin damage leads to the release of MBP into the bloodstream, resulting in elevated serum levels. The concentration of MBP in the blood serves as an indicator of central nervous system injury. Studies have shown that serum MBP levels in patients with AIS are positively correlated with infarct volume, suggesting its potential as a biomarker for brain injury assessment (58).

### 3.5 Exosomes and their circular RNA

Exosomes are nanoscale extracellular vesicles secreted by most cells, capable of crossing the blood–brain barrier and transferring various bioactive molecules between cells. They facilitate intercellular communication and are closely associated with the occurrence and progression of various diseases. Exosomes, particularly the functional substances they carry, play a crucial role in the pathogenesis and recovery process of ischemic stroke by affecting the neurovascular unit. Following an ischemic stroke event, various types of cells, including peripheral blood cells, endothelial cells, and brain cells, release exosomes. These exosomes can traverse the blood–brain barrier and be detected in cerebrospinal fluid and peripheral blood. Consequently, exosomes are increasingly recognized as potential biomarkers for the early diagnosis and prognosis of IS.

Xu et al. demonstrated that long non-coding RNAs (lncRNAs) such as lnc-CRKL-2, lnc-NTRK3-4, RPS6KA2-AS1, and lnc-CALM1-7, isolated from the serum of acute stroke patients, are significantly elevated (59). Bai et al. isolated exosomes from serum samples of IS patients and normal controls, finding elevated expression of circFUNDC1 in exosomes derived from IS patients’ serum. Receiver operating characteristic (ROC) analysis revealed an area under the curve (AUC) of 0.882 for circFUNDC1, indicating its high sensitivity and specificity as a diagnostic biomarker for IS (60). Xiao et al., through exosome circular RNA sequencing, large-sample validation, and diagnostic model construction, identified exosomal circ-0043837 and circ-0001801 as independent predictors of large-artery atherosclerosis (LAA) stroke. These circular RNAs showed significantly higher expression levels compared to controls, with diagnostic accuracies of AUC = 0.89 and AUC = 0.91, respectively, surpassing the diagnostic performance of plasma circular RNAs (61). Zhou et al. found that levels of miR-134 and miR-223 in exosomes from IS patients were significantly higher than those in non-ischemic stroke patients. Additionally, these levels correlated positively with NIHSS scores (*r* = 0.65, *p* < 0.01) and infarct volume (*r* = 0.68, *p* < 0.01), suggesting that miR-134 and miR-223 have potential diagnostic value for assessing the occurrence and severity of IS (62).

Exosomes are not only useful for the early diagnosis of IS but also help distinguish between different stages of the disease. Wang et al. found that, compared to controls, plasma exosomes in subacute and recovery-phase stroke patients showed significantly elevated levels of miRNA-21-5p, while exosomal miR-30a-5p was significantly higher in ultra-early stroke patients but lower than in controls during the acute phase. Furthermore, early diagnosis of large-artery atherosclerosis (LAA), which is associated with the worst prognosis, is particularly crucial (63). Zhang et al. observed significant increases in exosomal lnc_000048, lnc_001350, and lnc_016442 in LAA patients, with levels rising with stroke severity and showing better predictive capability for prognosis than NIHSS scores (64).

In recent years, multiple studies have demonstrated significant alterations in the expression of specific exosomal miRNAs in patients with AIS. Several miRNAs exhibit upregulated expression in AIS, including exosomal miR-212/132, miR-21, miR-9, miR-124, miR-134, and miR-223. In contrast, the expression level of exosomal miR-126 is downregulated in AIS patients (62, 65–68). These distinct miRNA expression patterns not only provide insights into the pathophysiological mechanisms of AIS but also hold potential clinical value as biomarkers for early diagnosis and disease assessment.

### 3.6 Other biomolecules

NETs are web-like structures released by neutrophils, primarily composed of free DNA, nucleosomes, and citrullinated histone H3 (citH3). NETs play a crucial role in the onset and progression of AIS. Studies have shown that NETs exacerbate early blood–brain barrier (BBB) disruption in AIS, increasing its permeability and potentially facilitating inflammatory cell infiltration, thereby aggravating neuronal damage (69). Research by Vallés et al. revealed that plasma NET levels in AIS patients were significantly higher than those in healthy individuals (70). Similarly, Lim et al. reported a marked increase in NET levels in AIS patients, and receiver operating characteristic (ROC) curve analysis demonstrated that the area under the curve (AUC) for double-stranded DNA (dsDNA) in early AIS diagnosis reached 0.859, suggesting its potential as an early diagnostic biomarker for AIS (17). Beyond its diagnostic implications, NETs are also closely associated with AIS severity and prognosis. Studies indicate that NET levels in thrombi and peripheral blood can reflect stroke severity and effectively predict short-term patient outcomes (71). Additionally, NETs may influence treatment responses in AIS. Evidence suggests that NETs could serve as prognostic biomarkers for futile recanalization following intravenous thrombolysis or mechanical thrombectomy. Arnaud et al. found that NETs were universally present in AIS thrombi, with all patients exhibiting NET-containing clots. High NET content in thrombi was correlated with failed recanalization, prolonged procedure time, and poorer stroke outcomes as assessed by the National Institutes of Health Stroke Scale (NIHSS) and modified Rankin Scale (mRS) scores (72). Moreover, analysis by Chen et al. demonstrated that the enrichment of NETs affects thrombus mechanical properties, potentially influencing the success rate of mechanical thrombectomy. Lower NET levels were significantly associated with higher rates of initial vascular recanalization (73).

To provide a more intuitive overview of these biomarkers and their roles, we present a summary table outlining their specific functions and recent research progress in ischemic stroke (Table 1).

## 4 Detection methods for ischemic stroke-related biomarkers

**Figure 2:**
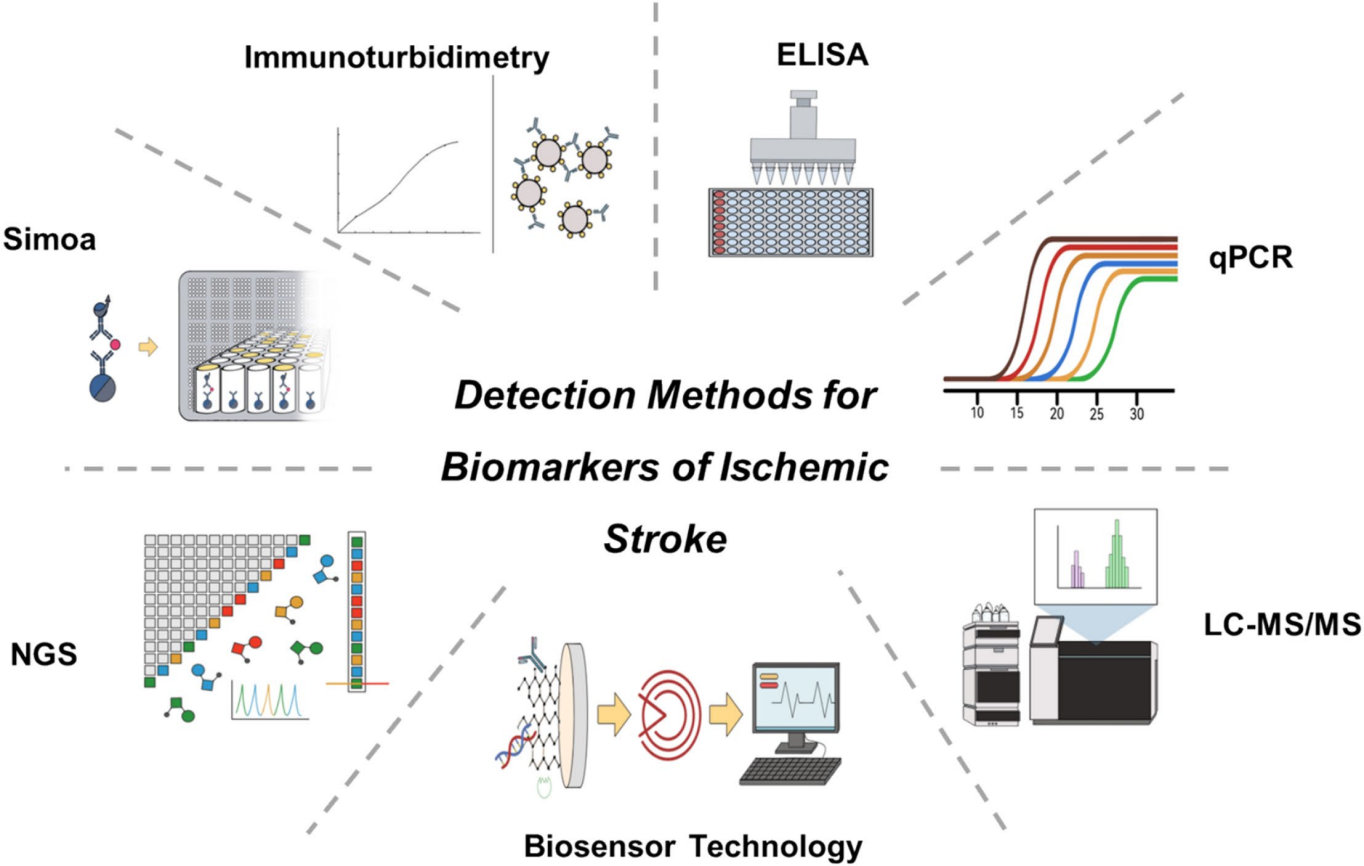
Common detection methods for ischemic stroke-related biomarkers.

Currently, common diagnostic methods for stroke biomarkers in clinical practice include enzyme-linked immunosorbent assay (ELISA), quantitative real-time PCR (qPCR), liquid chromatography-tandem mass spectrometry (LC-MS/MS), immunoturbidimetry, next-generation sequencing (NGS), single-molecule array (Simoa), and biosensing technologies (Figure 2). This paper summarizes the advantages, disadvantages, and clinical applications of various detection methods (Table 2).

**Table 2:** Comparison of detection methods for ischemic stroke-related biomarkers.

Detection method	Advantages	Limitations	Clinical significance	Clinical application status
ELISA	High sensitivity and specificity; simple operation; capable of detecting multiple biomarkers simultaneously.	Long detection time; high cost.	Commonly used for detecting inflammatory biomarkers such as CRP, IL-6, and TNF-α; suitable for large-scale studies and clinical validation.	Widely applied in clinical practice.
qPCR	High sensitivity; suitable for detecting low-abundance RNA molecules; accurate quantification.	High sample quality requirements; complex operation; relatively high cost.	Used for detecting genetic biomarkers such as miRNA and lncRNA; an essential tool in molecular biology research.	
LC–MS/MS	High sensitivity and specificity; capable of detecting multiple metabolites simultaneously.	Expensive equipment; complex operation; requires specialized technical support.	Widely used in metabolomics research (e.g., oxidative stress biomarker MDA detection); limited clinical translation.	
Immunoturbidimetry	Simple and rapid operation; relatively low cost.	Lower sensitivity and specificity.	Commonly used for rapid detection of coagulation and fibrinolysis system biomarkers (e.g., D-dimer); suitable for preliminary clinical screening.	
Simoa	Ultra-high sensitivity; simultaneous detection of multiple biomarkers; capable of detecting femtomolar concentrations.	High cost; requires specialized equipment.	Suitable for detecting neuronal injury biomarkers such as GFAP and NFL; plays an important role in early stroke diagnosis and prognosis assessment.	
NGS	High sensitivity and specificity; high throughput; automated; applicable to various biological samples.	High cost limits widespread application; complex data analysis; risk of false positives/negatives.	Used for detecting genetic mutations, vascular injury biomarkers, and epigenetic factors (e.g., miRNA, circRNA); facilitates precision diagnosis, classification, and novel biomarker discovery.	
Biosensors	High sensitivity; real-time detection; highly portable; suitable for point-of-care testing.	Susceptible to environmental interference; complex readout technology.	Has potential for on-site diagnosis of acute stroke; suitable for rapid detection of biomarkers such as GFAP.	Still in preclinical research stage.

### 4.1 Enzyme-linked immunosorbent assay

ELISA is a widely used method for detecting protein levels in body fluids, characterized by high sensitivity and specificity. It utilizes antigen–antibody reactions, with enzyme-labeled antibodies to detect target proteins. ELISA is suitable for detecting various stroke-related biomarkers, such as CRP (74–76), IL-6 (43, 74, 75), TNF-*α* (75, 76), ICAM-1, MMP-9 (43, 75), VCAM-1 (77, 78), GFAP^53^ (79, 80), and S100^53^ (81). The advantages of ELISA include simplicity of operation, good reproducibility, and the ability to detect multiple samples simultaneously. However, its disadvantages are longer detection time and higher costs.

### 4.2 Quantitative real-time PCR

qPCR is a technique used to quantify RNA levels by labeling PCR amplification products with fluorescent dyes and monitoring the fluorescence signal changes in real-time. It is suitable for detecting genetic biomarkers like miRNA and lncRNA. The advantages of qPCR include high sensitivity, strong specificity, and accurate quantification, capable of detecting low-abundance RNA molecules (82–84). However, qPCR requires high-quality RNA samples, is complex to operate, and has high costs.

### 4.3 Liquid chromatography-mass spectrometry

LC–MS/MS combines the separation capabilities of liquid chromatography with the high sensitivity of mass spectrometry. It is used to detect metabolites such as fatty acid derivatives, amino acids, and oxidative stress markers like 3-nitrotyrosine nitrated fibrinogen (85), 8-iso-PGF2α (86), and MDA (87). The advantages of LC–MS/MS are high sensitivity, strong specificity, and the ability to detect multiple metabolites simultaneously, making it a key tool in metabolomics research. However, LC–MS/MS equipment is expensive, the operation is complex, and it requires professional technicians.

### 4.4 Immunoturbidimetry

Immunoturbidimetry measures the turbidity changes in a solution due to antigen–antibody complex formation, quantifying protein biomarkers in blood. It is suitable for detecting coagulation and fibrinolysis system-related factors such as D-dimer, FIB, TAT, t-PAIC, APTT, PT, FDP, and SAA (88, 89). The advantages of immunoturbidimetry include rapid detection, ease of operation, and lower costs, but its sensitivity and specificity are relatively lower.

### 4.5 Next-generation sequencing

NGS is a high-throughput DNA sequencing technology used for comprehensive analysis of genomes, transcriptomes, and epigenomes. It can detect DNA methylation states and RNA expression profiles, suitable for studying complex genetic biomarkers (90, 91). The advantages of NGS include high throughput, strong sensitivity, and specificity, and the ability to detect thousands of genes and their regulatory elements simultaneously. However, NGS equipment and operating costs are high, data analysis is complex, and it requires professional bioinformatics support.

### 4.6 Single-molecule array

Simoa is a breakthrough in biomarker detection due to its extremely high sensitivity and specificity. This technology combines single-molecule immunocapture with fluorescence detection, capable of detecting extremely low concentrations of biomarkers, making it advantageous in stroke biomarker detection. Simoa’s core lies in its ability to capture and detect single target molecules in each reaction well, significantly enhancing detection sensitivity. Compared to traditional ELISA, Simoa can detect biomarkers at femtomolar levels. Simoa has been used to detect neuronal injury markers such as GFAP, NFL, and S100, which are rapidly released into the blood after brain tissue injury, providing crucial information for prognosis assessment (92–94).

Recent research has expanded the application of Simoa in stroke biomarker detection, developing multiplex detection platforms for simultaneous detection of multiple inflammatory and neuronal injury markers, improving detection efficiency and data reliability. Onatsu et al. utilized Simoa to analyze the NfL levels in 136 patients with AIS, discovering that the presence and extent of axonal injury estimated by NfL were correlated with the final infarct volume (95). Mattila et al. measured the GFAP levels and release rates in patients with acute cerebral ischemia using the Simoa method, finding that for patients with acute cerebral ischemia, prehospital sampling within 3 h combined with a specific rule (prehospital GFAP >410 pg./mL or prehospital GFAP 90–410 pg./mL combined with GFAP release >0.6 pg./mL/min) exhibited high specificity (NPV 98.4%) in 68% of acute cerebral ischemia patients (96).

### 4.7 Advances in biosensing technologies for stroke biomarker detection

Recent years have seen significant progress in the application of biosensing technologies for stroke biomarker detection, showing great potential and prospects. Biosensors combine biological recognition elements with physical sensors, converting biological molecules into detectable signals, enabling high sensitivity, rapid, and on-site detection, playing a key role in early diagnosis, condition monitoring, and personalized treatment of stroke.

#### 4.7.1 Electrochemical biosensors

Electrochemical biosensors detect biological molecules’ interactions with electrode surfaces to produce electrical signals, achieving high sensitivity detection. Rodríguez-Penedo et al. (97) developed a method for on-site GFAP detection using microcentrifuge tubes through electrochemical means, enabling rapid detection of hemorrhagic stroke biomarkers. These electrochemical biosensors provide high accuracy results in a short time, suitable for on-site acute stroke diagnosis.

#### 4.7.2 Optical biosensors

Optical biosensors leverage optical signal transduction mechanisms to enhance the sensitivity and specificity of biomarker detection. These sensors employ fluorescence detection, surface plasmon resonance (SPR), or colorimetric analysis to convert biomolecular interactions into quantifiable optical signals, significantly improving detection accuracy and real-time monitoring capabilities. Due to their high sensitivity and rapid response characteristics, optical biosensors are particularly suitable for early diagnosis of ischemic stroke and continuous biomarker monitoring.

#### 4.7.3 Microfluidic biosensors

Microfluidic biosensors use microfluidic technology to achieve biomarker detection. Sayad et al. developed a magneto-impedance-based microfluidic platform for detecting GFAP in blood, classifying acute stroke subtypes (98). This platform integrates microfluidic technology and magneto-impedance biosensors, providing high sensitivity and specificity in GFAP detection, offering a new method for early stroke diagnosis and classification.

#### 4.7.4 Comparison of biosensing technologies for AIS

To provide a clearer comparison of biosensing technologies used for detecting biomarkers in AIS, we have summarized the detection mechanisms, readout methods, and diagnostic performance of various biosensors (Table 3).

**Table 3:** Comparison of biosensors for AIS diagnosis.

Sensor	Biomarker	Detection range	Limit of detection (LOD)	Core mechanism	References
Electrochemical biosensors	CRP	0.01–5.0 μg/mL	0.008 μg/mL	Dual Magnetic Antibody Capture	(99)
	GFAP	10–1,000 pg/mL	3 pg/mL	Antibody Capture	(100)
	S100β	0.05–1 ng/mL	0.35 pg/mL	Au@AgNPs-Modified Antibody Capture	(101)
	Optical biosensors	CRP	0.889–20.7 μg/mL	1.2 μg/mL	Aptamer-mediated gold nanoparticle (AuNP) aggregation	(102)
GFAP		1 pg/mL to 50 ng/mL	1 pg/mL	Thionin acetate as a Raman reporter gene, AuNRs as a SERS probe	(103)
MMP-9		0.05-20 μg/mL	0.05 ng/mL	Optical Interference-Free Surface-Enhanced Raman Scattering CO-Nanotags	(104)
TNF-α/GFAP		/	0.023 pg/mL; 0.018 pg/mL	Gold nanorod array substrate based on surface-enhanced Raman scattering	(105)
TNF-α		/	1 pg/mL	A magnetic bead pull-down assay with purified and highly Raman-active gold nanoparticle clusters	(106)
MMP-9, IL-6, GFAP, IL-1β, TNF-α		/	0.21 pg/mL, 0.153 pg/mL, 0.106 pg/mL, 0.125 pg/mL, 0.15 pg/mL	5,5′-dithiobis-2-nitrobenzoic acid (DTNB) antibody-modified gold nanoparticles (AuNPs) on SERS devices as SERS probes	(107)
Microfluidic biosensors	GFAP	0.15–0.59 ng/mL	0.01 ng/mL	Magnetic labeling to capture GFAP	(98)
	CRP	/	0.1–50 mg/L	Integrated with Field-effect transistor (FET) sensor	(108)
	IL-6, GFAP, IL-8	/	437 pg/mL; 125 pg/mL; 2 pg/mL	Microbead-Based Quantum Dot-Linked Immunosorbent Assay	(109)

## 5 Conclusion

Biomarkers play a crucial role in the diagnosis, prognostic assessment, and therapeutic monitoring of ischemic stroke. However, despite extensive research identifying numerous stroke-related biomarkers, their clinical application remains limited by several challenges, including insufficient specificity, complex dynamic variations, labor-intensive detection methods, and a lack of standardization. An ideal stroke biomarker should possess characteristics similar to cardiac troponin T (cTnT) in myocardial infarction—accurately reflecting pathophysiological changes while being detectable through rapid, efficient, and precise methods suitable for clinical use.

Currently, ischemic stroke biomarkers primarily include inflammatory factors, neuronal injury proteins, and coagulation and fibrinolysis-related factors. However, these biomarkers typically represent only specific aspects of the pathological process rather than the entire disease course. Additionally, individual variations in stroke etiology limit the clinical applicability of single biomarkers. One promising future research direction is the integration of multi-omics data to identify core biomarkers that dynamically reflect stroke progression. Furthermore, the development of multiplex biomarker panels combining inflammatory, coagulation, neuronal injury, and metabolic markers may enhance diagnostic sensitivity and specificity. Incorporating imaging modalities and clinical scoring systems, such as the National Institutes of Health Stroke Scale (NIHSS), could further improve the clinical utility of biomarkers.

Beyond biomarker discovery, advancements in detection technologies will be critical for their successful clinical implementation. Current methodologies, including ELISA, qPCR, and LC–MS/MS, are widely used in research but face challenges such as complexity, lengthy processing times, and high equipment requirements, making them less suitable for the rapid diagnosis of this time-sensitive condition. In recent years, ultra-sensitive detection technologies, such as Simoa and biosensors, have achieved significant breakthroughs, with progressively lower detection limits. If these platforms can be further optimized to meet the needs of portable and point-of-care testing (POCT), the clinical translation of stroke biomarkers could be significantly accelerated. Additionally, the integration of artificial intelligence (AI) and machine learning algorithms may enhance diagnostic accuracy.

At present, most stroke biomarkers remain in the clinical research phase and have yet to be widely implemented in routine diagnostics. Future studies should focus on large-scale, multicenter cohort investigations with rigorous study designs, including prospective cohort studies, to establish clear reference ranges, specificity, and clinically relevant cutoff values for various biomarkers.

Overall, only by simultaneously advancing our understanding of the pathogenesis of AIS and enhancing the sensitivity, specificity, stability, and resistance to interference of detection technologies can stroke biomarkers truly support early diagnosis, disease monitoring, and personalized treatment strategies.
